# Effect of Forefoot Strike on Lower Extremity Muscle Activity and Knee Joint Angle During Cutting in Female Team Handball Players

**DOI:** 10.1186/s40798-016-0056-x

**Published:** 2016-08-15

**Authors:** Naruto Yoshida, Shun Kunugi, Sonoko Mashimo, Yoshihiro Okuma, Akihiko Masunari, Shogo Miyazaki, Tatsuya Hisajima, Shumpei Miyakawa

**Affiliations:** 1 Faculty of Health Care, Department of Acupuncture and Moxibustion, Teikyo Heisei University, 2-51-4 Higashi-ikebukuro, Toshima-ku, Tokyo, 170-8445 Japan; 2 Division of Sports Medicine, Graduate School of Comprehensive Human Sciences, University of Tsukuba, Ibaraki, Japan

**Keywords:** Knee valgus, ACL injury, Prevention, EMG, Motion analysis

## Abstract

**Background:**

The purpose of this study is to examine the effects of different strike forms, during cutting, on knee joint angle and lower limb muscle activity.

**Methods:**

Surface electromyography was used to measure muscle activity in individuals performing cutting manoeuvres involving either rearfoot strikes (RFS) or forefoot strikes (FFS). Three-dimensional motion analysis was used to calculate changes in knee angles, during cutting, and to determine the relationship between muscle activity and knee joint angle. Force plates were synchronized with electromyography measurements to compare muscle activity immediately before and after foot strike.

**Results:**

The valgus angle tends to be smaller during FFS cutting than during RFS cutting. Just prior to ground contact, biceps femoris, semitendinosus, and lateral head of the gastrocnemius muscle activities were significantly greater during FFS cutting than during RFS cutting; tibialis anterior muscle activity was greater during RFS cutting. Immediately after ground contact, biceps femoris and lateral head of the gastrocnemius muscle activities were significantly greater during FFS cutting than during RFS cutting; tibialis anterior muscle activity was significantly lower during FFS cutting.

**Conclusions:**

The results of the present study suggest that the hamstrings demonstrate greater activity, immediately after foot strike, during FFS cutting than during RFS cutting. Thus, FFS cutting may involve a lower risk of anterior cruciate ligament injury than does RFS cutting.

## Key Points

- We examined the differences in knee joint angles and muscle activities during forefoot strike cutting and rearfoot strike cutting.
- Forefoot strike cutting demonstrated significantly greater hamstring muscle activity and a tendency towards a lower knee valgus angle.
- Changing rearfoot strike cutting to forefoot strike cutting may reduce anterior cruciate ligament injury

## Background

Anterior cruciate ligament (ACL) injuries are common among female athletes and typically require at least 6–9 months of rehabilitation before athletic activity can be resumed. Athletes are often forced to reduce their level of competition following ACL injuries, making the prevention of ACL injuries a critical concern. Approximately 70 % of ACL injuries occur in non-contact situations, such as jump landing or stopping [1, 2], with a number of factors considered to be responsible. The hamstrings are muscles that, like the ACL, regulate the anterior movement of the tibia; therefore, weakness in or delayed mobilization of the hamstrings may result in susceptibility to ACL injury [3].

In handball, players may take up to three steps after catching the ball, eliciting the use of a variety of stepping techniques, depending on the situation [4]. Thus, playing handball requires advanced and varied movements; however, cutting, jump landing, and other quick movements may result in ACL injuries [5]. To prevent ACL injuries, handball players may need to practise steps that do not induce excessive knee valgus angles during play. Performing steps that do not invite injury requires consideration of foot strike forms, similar to the situation for marathon runners, in whom midfoot and forefoot strikes (FFS) reportedly improve running efficiency and potentially reduce injuries [6–8]. In addition, runners who employ FFSs are reported to have lower risks of knee and hip injuries than those using rearfoot strikes (RFS) [9]. In a comparison of FFSs and RFSs during running, Lieberman et al. [10] demonstrated differences in the amount of ground reaction force (GRF) absorbed by the body. They reported that runners who use FFSs generate smaller collision forces than do those using RFSs. This difference results primarily from a more plantarflexed foot, at landing, and more ankle compliance, during impact; this decreases the effective mass of the body that collides with the ground.

A previous study focused on frontal cutting [11] assumed that subjects with excessive knee valgus moments during cutting could make cutting easier by striking the foot more laterally. Another reported that selective activation of the medial/lateral and internal/external rotation muscles and co-contraction of the flexors and extensors helped to stabilize the joint [12]. To reduce maximum knee abduction moments, reducing either the GRF magnitude or its frontal plane moment arm is necessary [13]. Thus, reducing knee abduction moments has been achieved [14, 15] by instructing players to perform cuts with the stance foot closer to the midline and the trunk more erect. Although foot position and stance width [16] have been investigated, the effect of foot strike position differences during cutting on the knee’s valgus angle has not been explored. We believe that understanding this effect will be useful for determining effective ACL injury prevention steps. Thus, the objective of the present study was to examine the relationship between lower limb muscle activity and knee valgus angles during FFS and RFS cutting.

## Methods

### Research Participants

Eleven female handball players (mean age, 21.5 ± 0.9 years; mean height, 163.1 ± 5.2 cm; mean weight, 57.8 ± 3.8 kg) participated in the present study, which was approved by the University of Tsukuba Physical Education Research Institutional Review Board (Task No. PE 24-82). Each participant provided written or oral consent after receiving a thorough explanation of the study. All procedures followed during the conduct of this study were in accordance with the Declaration of Helsinki. We gave a direct advertisement to the female handball players in the Kanto College Handball League and recruited a sample of volunteers. Players were excluded from participating if they had any injury-related pain or disorder.

### Experiment Protocol

Surface electromyography was used to record lower limb muscle activity while participants performed FFS and RFS cutting. We calculated knee angle changes using a three-dimensional motion analysis, which permitted us to examine the relationship between muscle activity and knee joint angle. A force plate was synchronized with electromyography to compare muscle activity immediately before and after foot strike.

### Task

Each participant stood 80 cm to one side of the force plate and began moving towards it. After striking the centre of the force plate with one foot, the participant cut in a direction 60° lateral to the strike foot, relative to the direction of movement; the other foot then reached the goal mark (Fig. 1). Each participant was instructed to conduct this cutting movement as quickly as possible. A carpet was spread on the floor for safety. Each participant performed three trials; the average of the trials was calculated prior to analysis.

**Fig. 1:**
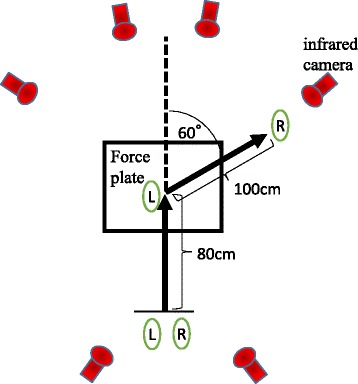
Cutting manoeuvre diagram (when the cutting with left leg). Each participant stood 80 cm to one side of the force plate. After striking the centre of the force plate with left foot, the right foot reached the goal mark

### Surface Electromyography

Muscle activity was recorded using surface electromyography (K800, Biometrics, Newport, UK) for the rectus femoris (RF), vastus medialis (VM), vastus lateralis (VL), semitendinosus (ST), biceps femoris (BF), tibialis anterior (TA), peroneus longus (PL), and the lateral head of the gastrocnemius (GL). Electromyography amplifiers (SX230-1000, Biometrics; inter-electrode distance 2 cm), which function as combined surface electrodes and amplifiers, were placed parallel to the direction of the muscle fibres; a grounding electrode was placed on the left wrist. Induced myogenic potential was converted to a digital display (sampling rate, 1000 Hz) and saved on a personal computer using the TRIAS data importing and analysis system (Biometrics). Raw electromyography signals were converted to root mean square values and used to compare strike forms. To examine muscle activity prior to and early in the foot strike, we determined muscle activity 50 ms prior to and 50 ms after toe contact with the strike plate.

### Assessment of Knee Valgus and Lower Leg External Rotation Angles

To assess knee and lower leg angles, we used a three-dimensional motion analysis system (Motive:Tracker; OptiTrack, Corvallis, OR, USA), a force plate (9268B; Kistler Group, Winterthur, Switzerland), and six infrared cameras (V100:R2; OptiTrack) with a sampling rate of 100 Hz. The jump landing point was on the force plate; GRF data were measured (sampling rate of 1000 Hz) and used to calculate the GRF at the jump landing point. Referring to a report by Kifuji [17], we placed reflective markers (diameter, 14 mm) on the tips of each participant’s shoes, lateral malleolus, inside and outside margins of the knee, greater trochanter, and anterior superior iliac spine. The inside and outside margins of the knee were defined based on the midpoint of their anteroposterior diameter, except the patella, which was defined at the height of the knee joint space. The centre of the hip joint was defined as the point one third of the distance from the greater trochanter on the line connecting the greater trochanter with the anterior superior iliac spine.

In the left femur coordinate system, the vector from the anterior superior iliac spine towards the midpoint between the outside and inside of the knee (knee midpoint, MK) was defined as LFz. The vector obtained from the cross product of the vector from the centre of the knee towards the outside of the knee and LFz was defined as LFx, and the vector obtained from the cross product of LFz and LFx was defined as LFy. In the left lower leg coordinate system, the vector from MK towards the midpoint between the medial malleolus and lateral malleolus (ankle midpoint, MA) was defined as LTz. The vector obtained from the cross product of the vector from the medial malleolus towards the lateral malleolus and LTz was defined as LTx, and the vector obtained from the cross product of LTz and LTx was defined as LTy. In the right femur coordinate system, the vector from the MK towards the anterior superior iliac spine was defined as RFz and the vector obtained from the cross product of the vector from the outside to the inside of the knee and RFz was defined as RFx; the vector obtained from the cross product of RFz and RFx was defined as RFy. In the right lower leg coordinate system, the vector from MK towards MA was defined as RTz; the vector obtained from the cross product of the vector from the lateral malleolus to the medial malleolus and RTz was defined as RTx, and the vector obtained from the cross product of RTz and RTx was defined as RTy. The left (right) knee angle was defined as the Euler angle in conversion from the left (right) femur coordinate system to the left (right) lower leg coordinate system using the rotation order (*Y*-axis → *X*-axis → *Z*-axis). These angles were used to calculate the knee valgus and lower leg rotation angles.

### Statistical Analysis

Data are represented as medians (25 and 75 %). To compare RFS and FFS cutting, we used SPSS Statistics for Windows, Version 19 (IBM, Armonk, NY, USA), to perform the Wilcoxon signed-rank test; the level of statistical significance was set as *p* < 0.05. We also calculated the effect size (*r* = *z* value/sqrt (number of samples) as part of the post hoc power analysis.

## Results

No significant differences were observed in peak knee flexion angles, rotation angles, or GRF peaks between FFS and RFS cutting (Tables 1 and 2). However, the valgus angle tends to be smaller during FFS cutting than during RFS cutting (*p* = 0.06). Just prior to ground contact (50 ms before), BF (*p* = 0.008, *r* = 0.805), ST (*p* = 0.033, *r* = 0.644), and GL (*p* = 0.003, *r* = 0.885) muscle activities were significantly greater during FFS cutting than during RFS cutting; TA muscle activity was greater (*p* = 0.010, *r* = 0.778) during RFS cutting (Table 3). Immediately (50 ms) after ground contact, BF (*p* = 0.003, *r* = 0.885) and GL (*p* = 0.003, *r* = 0.885) muscle activities were significantly greater during FFS cutting than during RFS cutting; TA muscle activity was significantly lower (*p* = 0.004, *r* = 0.885) during FFS cutting (Table 4).

**Table 1:** Knee joint angles (degrees)

	Flexion		Internal rotation		Valgus*	
	RFS	FFS	RFS	FFS	RFS	FFS
Max	61	64	3	5	5	6
75 %	56	55	−13	−12	4	3
Median	52	50	−16	−15	3	2
25 %	49	49	−19	−22	2	0
Min	46	41	−25	−26	−5	−7

*FFS* forefoot strike cutting, *RFS* rearfoot strike cutting

*RFS vs FFS, *p* = 0.06

**Table 2:** Ground reaction forces

	RFS (Newtons)	FFS (Newtons)
Max	1392.5	1491.1
75 %	1225.2	1205.3
Median	1165.7	1160.8
25 %	1048.5	1091.5
Min	877.2	891.9

Significant differences were not observed between FFS and RFS cutting

*FFS* forefoot strike cutting, *RFS* rearfoot strike cutting

**Table 3:** Muscle activity 50 ms before foot strike (%)

	VM		RF		VL		ST*		BF*		TA*		GL*		PL	
	RFS	FFS	RFS	FFS	RFS	FFS	RFS	FFS	RFS	FFS	RFS	FFS	RFS	FFS	RFS	FFS
Max	239	293	36	48	134	136	56	67	36	90	77	44	68	91	38	60
75 %	63	61	17	15	42	60	29	38	21	42	53	29	27	46	28	33
Median	24	36	8	8	25	29	24	30	16	24	47	25	20	38	20	24
25 %	15	19	5	5	23	23	19	25	12	13	43	16	16	32	12	14
Min	8	11	4	4	14	14	10	14	7	12	19	7	12	16	7	7
Effect size (*r*)	0.349		0.108		0.027		0.644		0.805		0.778		0.885		0.081	

BF, ST, and GL muscle activities were significantly greater during FFS than during RFS cutting, and TA muscle activity was significantly greater during RFS cutting

*RF* rectus femoris, *VM* vastus medialis, *VL* vastus lateralis, *ST* semitendinosus muscle, *BF* biceps femoris, *TA* tibialis anterior, *GL* lateral head of the gastrocnemius, *PL* peroneus longus, *FFS* forefoot strike cutting, *RFS* rearfoot strike cutting

*RFS vs FFS, *p* < 0.05

**Table 4:** Muscle activity 50 ms after foot strike (%)

	VM		RF		VL		ST		BF*		TA*		GL*		PL	
	RFS	FFS	RFS	FFS	RFS	FFS	RFS	FFS	RFS	FFS	RFS	FFS	RFS	FFS	RFS	FFS
Max	255	299	52	62	144	141	121	112	62	116	123	61	79	160	52	67
75 %	66	63	25	18	45	61	53	63	41	65	85	31	34	85	41	48
Median	24	37	10	9	34	32	45	55	30	49	77	28	31	70	28	33
25 %	20	25	8	7	26	28	34	45	21	33	66	23	23	57	19	19
Min	10	12	6	6	16	16	22	26	18	27	40	14	16	25	11	12
Effect size (*r*)	0.376		0.349		0.322		0.376		0.885		0.858		0.885		0.081	

BF and GL muscle activities were significantly greater during FFS than during RFS cutting; TA muscle activity was significantly lower during FFS cutting

*RF* rectus femoris, *VM* vastus medialis, *VL* vastus lateralis, *ST* semitendinosus muscle, *BF* biceps femoris, *TA* tibialis anterior, *GL* lateral head of the gastrocnemius, *PL* peroneus longus, *FFS* forefoot strike cutting, *RFS* rearfoot strike cutting

*RFS vs FFS, *p* < 0.05

## Discussion

To examine differences in knee joint stress between RFS and FFS cutting, we compared the two forms of cutting with respect to peak flexion, valgus, and external rotation angles. Although no significant differences were observed in peak flexion or external rotation angles, there was a tendency for the peak valgus angle to be smaller during FFS cutting than during RFS cutting (*p* = 0.06). The “position of no return,” as reported by Ireland [18], is the dynamic alignment that may easily lead to ACL injury. At that point of hip adduction and internal rotation, the knee is less flexed and valgus; knee valgus is a recognized risk factor for ACL trauma. In the current study, knee valgus during FFS cutting is less than that during RFS; therefore, FFS might be a technique for preventing ACL injury.

GL muscle activity 50 ms prior to ground contact was greater during FFS cutting than during RFS cutting, demonstrating that muscle activity during plantar flexion of the foot was high prior to ground contact. In addition, pre-activation for eccentric contraction of the GL may have occurred to absorb the impending impact during ground contact. After ground contact, GL action was high, but TA muscle activity was low. FFS cutting simultaneously results in increased GL muscle activity and inhibited TA muscle activity. Anterior movement of the tibia decreases by 40 % during ankle joint plantarflexion compared with that during dorsiflexion, contributing to knee joint stability [19]. This suggests that increasing GL muscle activity during FFS might contribute to a decreased risk of ACL injury.

An intriguing finding of the present study is that BF muscle activity also increased with GL muscle activity, 50 ms after ground contact. A previous study reported that ACL injuries occur within 50 ms of ground contact [20], demonstrating the importance of impact absorption. Regardless of the similarities in peak GRFs, the significant differences in knee valgus angles indicate that the vertical impact force occurring during RFS cutting may be absorbed by knee valgus. In a comparison of FFS and RFS forms during running, knee valgus moments demonstrated significantly lower values during FFS [21]; this finding supports the present results.

The high BF and ST muscle activities during toe contact may have been evoked by the plantarflexion of the talocrural joint, which is sometimes emphasized during gait. In this gait pattern, the body’s centre of gravity is moved forward, resulting in early heel off and shifting of the line of the GRF action forward. Therefore, a gait occurs in which the ankle plantarflexes and the knee flexes [22]. Based on the expectation that BF activity increases to flex the knee joint, knee flexion with ankle plantarflexion may also be induced by the same mechanism during toe cutting. MacWilliams et al. reported that hamstring strengthening may reduce ACL injury by reducing the load on the ligament [3]. Mette et al. also reported that non-injured female athletes with reduced ST preactivity during side cutting had increased risks of future non-contact ACL ruptures [23]. These findings support the results of the present study. As previously stated, hamstring weakness or delayed mobilization may be key factors in ACL injury; thus, when performing single-leg landing or cutting, faster and more powerful hamstring activity may contribute to reduced risk of ACL injury. Co-activation of the quadriceps femoris muscles and hamstrings increases articular pressure and enhances knee stability [24]. However, solo contraction of the quadriceps femoris muscles increases ACL tension [25, 26]. Thus, during co-activation, hamstring enhancement that is simultaneous with activation of the quadriceps femoris muscles is important. In the present study, increased BF and ST activities during toe contact suggest that toe contact may inhibit ACL injury.

In a video analysis, Olsen et al. proposed that knee valgus during near-full knee extension is a common characteristic of handball-related injuries [27]. Therefore, during cutting with near-full knee extension, minimization of the knee valgus angle may be useful for preventing ACL injury. Another handball movement, the *stop feint*, requires players to quickly stop both feet and then feint to move left or right past an opponent. Stopping both feet using an RFS (heels contacting the ground) may increase the risk of knee injury.

These observations suggest that there are differences in hamstring and gastrocnemius muscle activities and in knee valgus angles between FFSs and RFSs. Therefore, we recommend that athletes practise the stop feint using FFS (toe contact). Authors investigating knee abduction torques during side cutting in handball players also recommended toe landings for prevention of ACL injuries [13]. Moreover, there were fewer toe landings associated with ACL ruptures. Toe landing improves the alignment of the leg and helps athletes avoid risky alignments that may lead to ACL injury [28]. The muscle activity results from the current study agree with those described for other related sports. Beyond developing quick and powerful hamstrings, the avoidance of heel strikes and incorporating toe-strike cutting is expected to reduce knee valgus angles and, in turn, prevent ACL injury.

Several limitations of the present study must be noted. First, the study sample size was small. Second, the participants included only female handball players, making it difficult to apply these results more broadly. In future research, an examination of joint torques will also be necessary. Further, investigating cutting and turning at angles and directions other than those used in the present study will be necessary.

## Conclusions

The hypothesis of this study was that FFS cutting involves greater hamstring activity and smaller knee valgus angles than does RFS cutting, placing less stress on the knees. Hamstring muscle activity immediately after ground contact was higher during FFS cutting than during RFS cutting, suggesting that FFS cutting, compared with RFS cutting, may reduce the risk of ACL injury.
